# gp130 Activates Mitochondrial Dynamics for Hepatocyte Survival in a Model of Steatohepatitis

**DOI:** 10.3390/biomedicines11020396

**Published:** 2023-01-29

**Authors:** Daria Shunkina, Anastasia Dakhnevich, Egor Shunkin, Olga Khaziakhmatova, Valeria Shupletsova, Maria Vulf, Alexandra Komar, Elena Kirienkova, Larisa Litvinova

**Affiliations:** Centre for Immunology and Cellular Biotechnology, Immanuel Kant Baltic Federal University, Kaliningrad 236041, Russia

**Keywords:** metabolic syndrome, non-alcoholic fatty liver disease (NAFLD), IL-6, gp130, mitochondria, oxidative stress, inflammation, metabolism

## Abstract

Obesity is the main cause of metabolic complications. Fatty liver infiltration is a companion of obesity. NAFLD is associated with impaired energy metabolism with an excess of nutrients. Mitochondrial dynamics are important for the regulation of energy balance, which regulates mitochondrial function, apoptosis, and mitophagy. The aim of this study was to investigate the effect of gp130 on the components of mitochondrial dynamics in a cellular model of steatohepatitis. Addition of IL-6/gp130 contributed to an increase in the percentage of live cells and a decrease in the percentage of dead and apoptotic cells. Addition of IL-6/gp130 increased the expression of NF-kB1 gene and mitochondrial dynamics markers (MFN2 and TFAM) in HepG2 with tBHP/Oleic. Addition of IL-6 or gp130 reduced the expression of cytoprotector genes (HSF1 and HSP70) in HepG2 cell cultures with tBHP/Oleic. Increased mitochondrial dynamics gene activity protected against HepG2 cell death in the steatohepatitis model. Trans-signaling resulted in increased TFAM and MAPLC3B, and decreased DNM1L gene expression in HepG2 with tBHP/Oleic.

## 1. Introduction

Obesity is the leading cause of metabolic complications [1]. With the worldwide increase in obesity, the prevalence of liver disease has also greatly increased [2] from 15% to 25% [3]. NAFLD is a common concomitant of obesity [2]. The etiology of NAFLD is the burden of high triglyceride and glucose levels on the liver without significant alcohol consumption, resulting in abnormal fat accumulation in the liver [4].

NAFLD includes a wide spectrum of diseases of varying severity, such as steatosis, steatohepatitis (NASH), and cirrhosis [5]. Hepatic steatosis is diagnosed when more than 5% of the liver mass is infiltrated with fat [6]. Fatty infiltration of the liver with the presence of inflammatory foci has been isolated to a separate disease NASH. Steatohepatitis is the most common complication of NAFLD. The progression of steatosis in NASH is likely multifactorial and involves insulin resistance, toxic lipid accumulation, and inflammation in the liver. NASH manifests as increasing liver fibrosis and carries a potential risk for the development of hepatocellular carcinoma [7].

Insulin resistance plays a key role in the development of NAFLD, as it causes increased hepatic lipogenesis and inhibition of lipolysis and inflammation of adipose tissue [8]. Inflammation due to fatty liver infiltration is maintained by cellular oxidative stress in insulin-dependent tissues [9]. The deposition of triglycerides in the liver triggers the processes of protein glycosylation and free radical substitution reactions. Free radicals disrupt the electron transport chain at the mitochondrial membrane and promote the formation of reactive oxygen species (ROS) [10]. Thus, disturbances of lipid metabolism in the liver affect mitochondria, which are ROS generators in cells. ROS damages proteins, DNA, and RNA. Mitochondria control ROS damage by removing defective proteins and repairing mtDNA. In addition, removal of defective mitochondria by mitophagy reduces the level of ROS under conditions of mild oxidative stress. Acute exposure of ROS requires mitochondrial fission or fusion [11]. ROS-reactive kinases can either inhibit/initiate mitochondrial fusion or stimulate mitochondrial fission by altering mitochondrial morphology.

Mitochondrial dynamics refers to the co-ordinated steps of fission and fusion by which mitochondria maintain their shape, distribution, and size [12]. Mitochondrial dynamics is important for the regulation of apoptosis, mitophagy, energy balance, immunity, and the maintenance of mitochondrial function [13]. Mitochondrial fusion can rescue mitochondria with mutations in various genes [14]. However, when mitochondria cannot be restored to a healthy state by these processes, they are removed by selective autophagy [15].

Mitochondrial function is adapted in NAFLD mainly by down-regulating the electron transport chain (ETC) and increasing the ability of mitochondria to oxidize fatty acids [16]. Meanwhile, more and more molecules related to mitochondrial dynamics have been identified [17]. Interleukin-6 (IL-6) affects mitochondrial dynamics. IL-6 is associated with hepatic lipid homeostasis in steatotic mice. When mice were fed a high-carbohydrate diet, exogenous plasma IL-6 counteracted the accumulation of neutral lipids in the liver, mitochondrial dysfunction, and hepatocyte death [18]. However, the cytoprotective effects of IL-6 have not yet been reliably confirmed.

IL-6 has pleiotropic effects as it acts on cells in several ways. IL-6 forms a complex with the membrane receptor IL-6R. IL-6R is expressed on hepatocytes and some leukocytes. IL-6/IL-6R binds to the second receptor protein, glycoprotein gp130 [19]. This activation method is referred to as classical signaling. However, a soluble form of the sIL-6R receptor also exists [20]. sIL-6R can be formed by partial proteolysis in response to oxidative stress [21]. The IL-6/IL-6R or IL-6/sIL-6R complex binds to gp130, which is expressed in all cells. This process is referred to as trans-signaling.

While gp130 is expressed in all cells, IL-6R is present only on hepatocytes and leukocytes. According to studies, trans-signaling activation is pro-inflammatory, while the classical type of IL-6 activation is required for the regenerative or anti-inflammatory effect of the cytokine in the liver [22].

In this context, the aim of this study was to investigate the effect of gp130 on the components of mitochondrial dynamics in a cellular model of steatohepatitis.

## 2. Materials and Methods

### 2.1. Cell Preparation

The HepG2 cell line was acquired from the CCP “Collection of Vertebrate Cell Cultures” of the Institute of Cytology of the Russian Academy of Sciences (St. Petersburg, Russia).

Two cell models were created: the oxidative stress model with the addition of tert-Butyl hydroperoxide (tBHP) and the steatohepatitis model with the addition of tBHP and oleic acid (Oleic). The initial solutions of oleic acid and tBHP were prepared in 75% alcohol and water. Dilution to the working concentration (0.5 mM) was performed in complete culture medium.

Cells (30 × 10^4^ cells/well) were grown in 6-well culture plates (Eppendorf, Hamburg, Germany) at 37 °C in a CO_2_ incubator in an atmosphere of 5% CO_2_ in Dulbecco’s Modified Eagle’s Medium (DMEM) (Capricorn Scientific, Ebsdorfergrund, Germany) containing 10% embryonic bovine serum (Capricorn Scientific, Ebsdorfergrund, Germany), 1% penicillin/streptomycin antibiotics (Capricorn Scientific, Ebsdorfergrund, Germany), 1% stable L-glutamine (Capricorn Scientific, Ebsdorfergrund, Germany), and 0.1% non-essential amino acids (Capricorn Scientific, Ebsdorfergrund, Germany) for 48 h until the cells reached 80% confluence. Then, the medium was replaced with high glucose DMEM (glucose content 4.5 g/L) and tBHP and oleic acid were added in the experimental models. For the oxidative stress model, DMEM high glucose was added for 22 h followed by tBHP for 2 h. For the steatohepatitis model, the tBHP solution was added 22 h after the start of cultivation with oleic acid and incubated for an additional 2 h. The total incubation time for the cells of each model was 24 h

Then, the medium was replaced by DMEM with high glucose with IL-6 (concentration of 7 ng/mL) and IL-6 with gp130 (concentration of 100 ng/mL). Samples were cultured for an additional 24 h and then removed from the wells with 0.02% Versin solution (Sigma-Aldrich, St. Louis, MO, USA) and 0.25% Trypsin solution (Sigma-Aldrich, St. Louis, MO, USA) for further analysis.

For each model, a positive control was prepared in which the cells were treated only with the reagents without further addition of proteins. All models were created in 12 repetitions. A negative control was also created without the addition of reagents and proteins.

### 2.2. Oil Red Staining

Cells were fixed with 4% paraformaldehyde solution for 30 min and then washed three times with PBS followed by propylene glycol. The fixed preparations were stained for 15 min with propylene glycol solution with the addition of 0.5% oil red (Sigma-Aldrich, St. Louis, MO, USA). Cells were washed with 60% propylene glycol and distilled water. Images of cells with stained lipid droplets were acquired with an inverted phase contrast microscope (INV100; BEL Engineering, Monza (MB), Italy) at 200× magnification and photographed with a Bel Photonics camera.

The area of a lipid droplet was calculated using a stereological instrument and a point counting method [23]. A grating mask with an area/dot value of 6518.5 nm was superimposed on the image of a lipid droplet (13,500×). Each grid point has the shape of a cross delineating the four quadrants. Lipid droplets located in the upper right quadrant of the grid points were counted. The areas were determined using the following equation: = ∑pa/p (is the area, ∑p is the sum of the counted points, a/p is the value of the area/point).

### 2.3. Evaluation of the Viability of Cellular Systems

The viability of the cellular systems was evaluated by flow cytofluorimetry. After calibration and adjustment of negative control settings, analysis was performed using GuavaViaCount (Guava ViaCount Reagent, Millipore, Burlington, AS, USA) according to the standard color protocol and analysis according to the manufacturer’s recommendations. We used the flow cytofluorimeter MACSQuant Analyzer 7 (Miltenyi Biotec, Bergisch Gladbach, Germany).

### 2.4. Analysis of gene expression

Total RNA from homogenized biopsies of AT was isolated using an ExtractRNA kit (Evrogen, Moscow, Russia). The resulting RNA was dissolved in 30 μL nuclease-free water. The purity and concentration of the isolated RNA was determined using a spectrophotometer (Nanovue Plus, GE Healthcare Bio-Sciences, Uppsala, Sweden). The quality of total RNA was determined by the RIN index (the RNA Integrity Number). Reverse transcription was performed using (dT) 23 (Beagle, St. Petersburg, Russia) and M-MLV reverse transcription (Evrogen, Moscow, Russia). To determine relative gene expression, qPCR was performed using qPCRmix- HS reagents (Evrogen, Moscow, Russia). We used 4 μL cDNA as template and RPLPO (a large ribosomal protein) as reference gene. Sequences of primers and probes for PCR (Table 1) are as follows.

### 2.5. Western Blotting Analysis

Protein lysates were obtained using RIPA buffer (BioRad, Hercules, CA, USA) and normalized using the Bradford method (Reagent Bradford BioRad buffer, BioRad, Hercules, CA, USA) with separation by polyacrylamide gel electrophoresis (BioRad, Hercules, CA, USA). Transfer of proteins was performed on a PVDF membrane at +4 °C for 2 h (BioRad, Hercules, CA, USA). Hybridization of target proteins was performed with the corresponding primary antibodies and secondary antibodies labeled with horseradish peroxidase (Thermo Fisher Scientific, Waltham, AS, USA) using a substrate for visualization of Western blots ECL Plus (Thermo Fisher Scientific, Waltham, AS, USA). Analysis of the staining intensity of the bands was performed in the ImageJ program with normalizations for GAPDH protein.

Incubation with primary antibodies was performed overnight at +4. Incubation with secondary antibodies conjugated with horseradish peroxidase (Thermo Fisher Scientific, Waltham, AS, USA) was performed for 2 h. Then, densitometry was measured on a ChemiDoc MP (Bio-Rad, Hercules, CA, USA) using a substrate for visualization of Western blots ECL Plus (Thermo Fisher Scientific, Waltham, AS, USA). The following primary antibodies were used: NFkB (701079, Invitrogen, Waltham, AS, USA), mitochondrial transcription factor A (TFAM) (MA5-16148, Thermo Fisher Scientific, Waltham, AS, USA), dynamin-1-like protein (DRP1) (OTI3F4, Invitrogen, Waltham, AS, USA), mitofusin 2 (MFN2) (MA5-27647, Invitrogen, Waltham, AS, USA), Bcl-2 Associated X-protein (BAX) (MA5-13994, Invitrogen, Waltham, AS, USA), HSPA1A heat shock protein family A (Hsp70) (MA3-006, Invitrogen, Waltham, AS, USA), superoxide dismutase type 2 (SOD2) (MA5-31514, Invitrogen, Waltham, AS, USA). All target proteins were normalized to glyceraldehyde-3-phosphate dehydrogenase (GAPDH) protein (ZG003, Thermo Fisher Scientific, Waltham, AS, USA).

### 2.6. Statistical Analysis

The normal distribution of the quantitative indicators was tested using the Kolmogorov–Smirnov test and the Shapiro–Wilk test. In the case of normal distribution, the hypothesis of equality of sample means was tested using the Student *t*-test. The nonparametric Kruskal–Wallace test was used to test the significance of differences between independent quantitative samples that do not follow the law of normal distribution. In the case of statistically significant differences between groups, the analysis was performed using the Mann–Whitney test. Differences were considered significant at a significance level of *p* < 0.05.

To determine relative gene expression, qPCR was performed using qPCRmix-HS reagents (Evrogen, Moscow, Russia). We used 4 μL cDNA as template and *RPLPO* (a large ribosomal protein) as reference gene. Gene expression levels were calculated using the delta-delta Ct method, also known as 2^−∆∆Ct^ formula, which was used to calculate the relative abundance of gene expression in the samples [24].

Band staining intensity analysis was performed using Image Lab software (Bio-Rad, Hercules, CA, USA) with normalizations for GAPDH.

The presence of a relationship between the studied parameters was determined using Spearman correlation. Statistical analyzes and plots were generated in GraphPad Prism 8.01 (GraphPad Software, Boston, AS, USA) from the initial data.

## 3. Results

### 3.1. Steatohepatitis Cell Cultures

**Figure 1:**
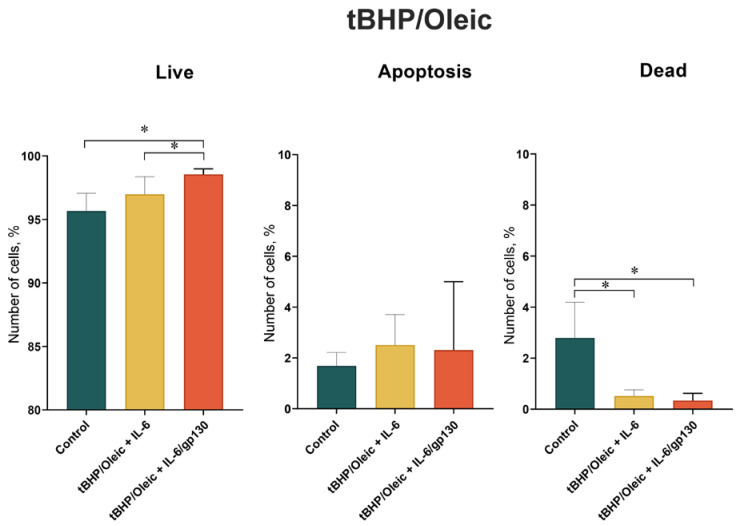
Percentage of live, dead, and apoptotic cells in HepG2 cultures with tBHP/Oleic; Mean ± SD; *—*p* < 0.05.

The HepG2 cell line was treated with tBHP/Oleic to create a model for oxidative stress and to create a model for steatohepatitis. The percentage of live cells in the HepG2 cell culture decreased when tBHP/Oleic was added (Figure 1). The percentage of dead and apoptotic cells in the HepG2 cell culture increased when tBHP/Oleic was added (Figure 1).

**Figure 2:**
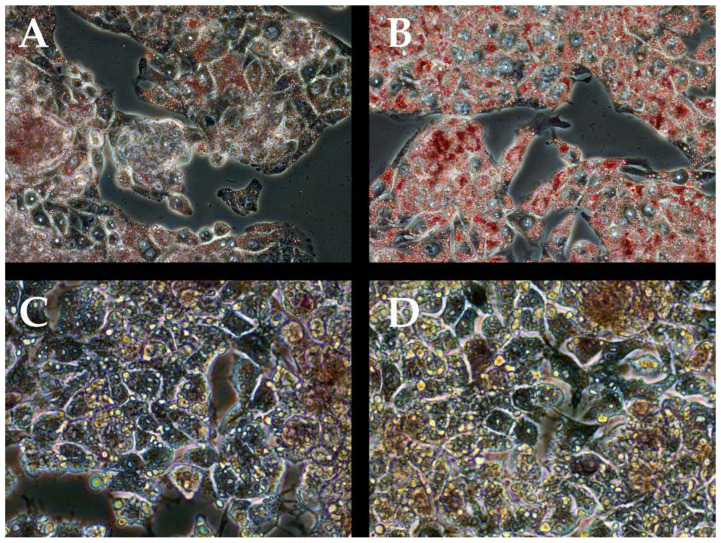
Relative areas of lipid droplets in HepG2 cell cultures. 0.5% oil red staining of HepG2 cell models (200× magnification). (**A**)—HepG2 control culture; (**B**)—HepG2 culture with addition of tBHP/Oleic; (**C**)—HepG2 culture with addition of tBHP/Oleic and IL-6 (oil red and hematoxylin staining); (**D**)—HepG2 culture with addition of tBHP/Oleic and IL-6/gp130 (oil red and hematoxylin staining).

The addition of Oleic increased the area of lipid inclusions in the HepG2 cell culture with the addition of tBHP/Oleic (Figure 2).

**Figure 3:**
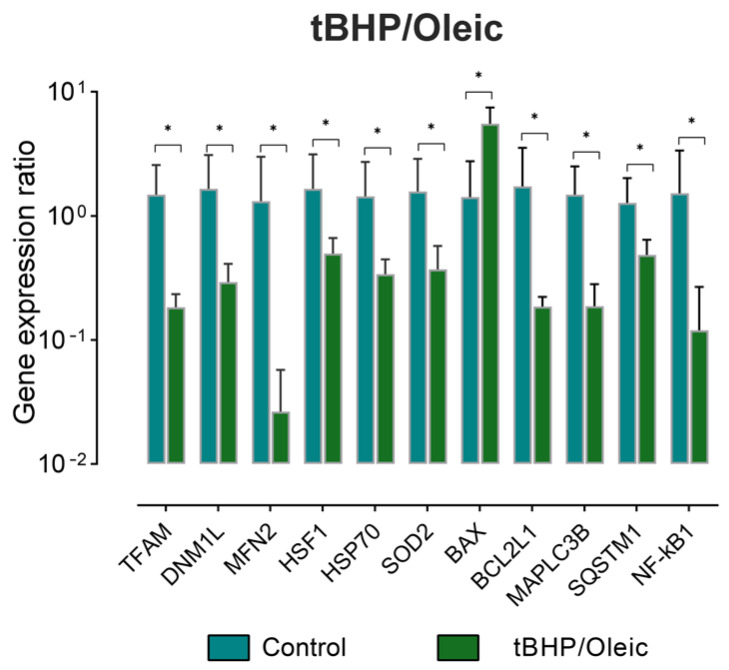
The level of gene expression in HepG2 cell model with the addition tBHP/Oleic. Mean ± SD; *—*p* < 0.05.

The expression of marker genes for mitochondrial dynamics (*TFAM, DNM1L, MFN2*), cytoprotectors associated with mitochondrial biogenesis (*HSF1, HSP70, SOD2*), apoptosis (*BAX, BCL2L1*), and mitophagy (*MAPLC3B, SQSTM1, NF-kB1*) was analyzed in the experimental model with the addition tBHP/Oleic acid (Figure 3). Expression of all analyzed genes, except *BAX*, decreased with tBHP/Oleic (Figure 3).

**Figure 4:**
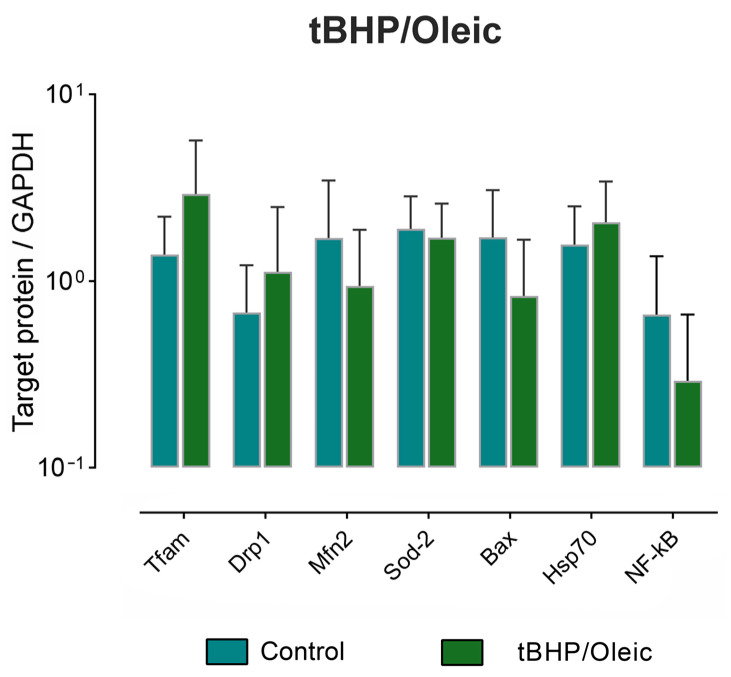
The level of protein production in HepG2 cell model with the addition of tBHP/Oleic; Mean ± SD.

The production of the most highly expressed marker proteins in the experimental model was analyzed: the production of mitochondrial dynamics proteins (Tfam, Dnm-1, Mfn-2), cytoprotectors associated with mitochondrial biogenesis (Hsp70, Sod-2), apoptosis (Bax, NF-kB1) (Figure 4). When adding tBHP/Oleic protein products were not changed (Figure 4).

### 3.2. IL-6 in Cell Model tBHP/Oleic

Addition of IL-6 increased the percentage of live cells in HepG2 cell culture with tBHP/Oleic. Addition of IL-6 decreased the percentage of dead and apoptotic cells in HepG2 cell culture with tBHP/Oleic (Figure 1).

**Figure 5:**
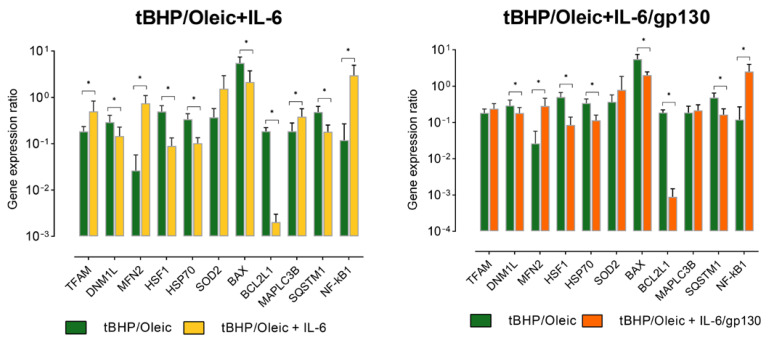
The level of gene expression in HepG2 cell models with the addition of IL-6 and IL-6/gp130; Mean ± SD; *—*p* < 0.05.

Addition of IL-6 increased the expression of *TFAM* and *MFN2* genes in HepG2 cell culture with tBHP/Oleic (Figure 5).

Addition of IL-6 decreased *DNM1L* gene expression in HepG2 cell cultures with the addition of tBHP/Oleic (Figure 5).

**Figure 6:**
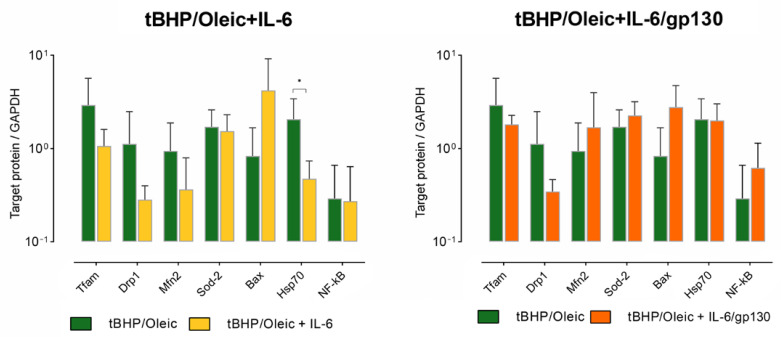
Protein production levels in HepG2 cell models with the addition of IL-6 and gp130. (Mean ± SD), *—*p* < 0.05.

Addition of IL-6 decreased *HSP70* gene expression in HepG2 cells with tBHP/Oleic (Figure 5). HSP70 protein decreased in HepG2 cells with tBHP/Oleic compared with the HepG2 control culture (Figure 6). *HSF1* gene expression decreased in HepG2 cell cultures with the addition of tBHP and tBHP/Oleic (Figure 5).

Addition of IL-6 decreased *HSP70* gene expression in HepG2 cells with tBHP/Oleic (Figure 5). *HSF1* gene expression decreased in HepG2 cells with the addition of tBHP/Oleic (Figure 5).

Protein production did not change in HepG2 cells with the addition of tBHP/Oleic (Figure 6).

### 3.3. Co-addition of IL-6 and gp130 in Cell Models with tBHP/Oleic

Addition of gp130 increased *MFN2* gene expression in HepG2 cells (Figure 5). Gp130 decreased *HSP70* gene expression in HepG2 cells with tBHP/Oleic (Figure 5). *HSF1* gene expression decreased in HepG2 cells with tBHP/Oleic (Figure 5).

Gp130 increased *NF-kB1* gene expression in HepG2 cells with tBHP/Oleic (Figure 5). Gp130 decreased *BAX* gene expression in HepG2 cells with tBHP/Oleic (Figure 5). *BCL2L1* gene expression decreased in HepG2 cells with tBHP/Oleic (Figure 5). *SQSTM1* gene expression decreased in HepG2 cells with tBHP/Oleic (Figure 5).

## 4. Discussion

Obesity is a risk factor for the development of fatty liver infiltration [25]. The formation of lipid droplets in hepatocytes is a sign of NAFLD [26]. Addition of tBHP/Oleic to HepG2 cell cultures stimulated an increase in the area of lipid inclusions in hepatocytes.

Numerous new molecules have been identified that influence local and systemic inflammatory imbalance in obesity [27]. IL-6 exhibits a dual character and may be a proinflammatory [28]. and anti-inflammatory cytokine [29]. IL-6 is an important inducer of acute phase response and protection against infection [30].

**Figure 7:**
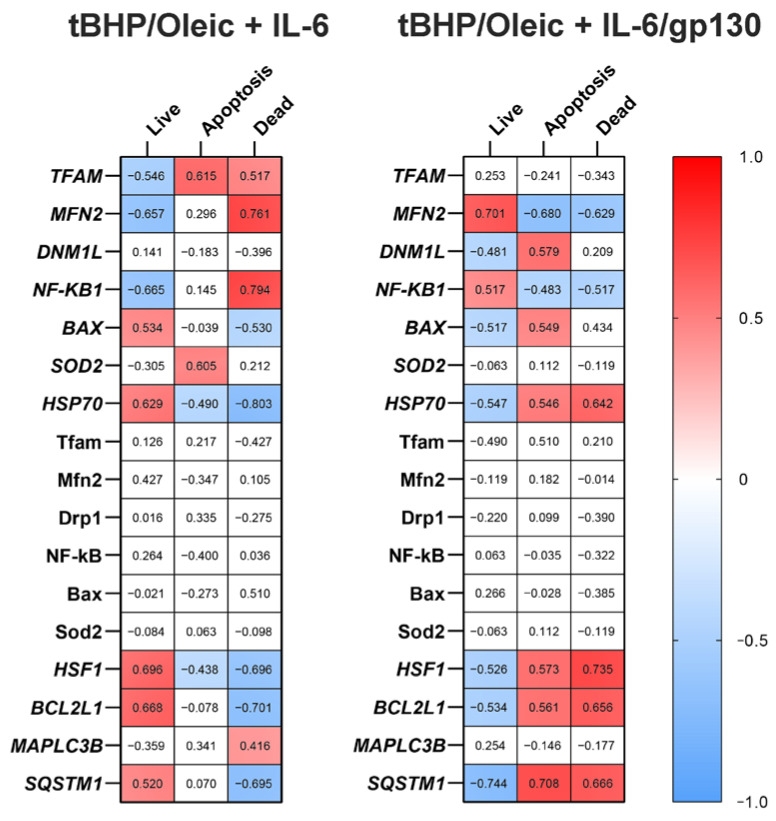
Correlations of the studied parameters with cell viability of the HepG2 cell model with tBHP/Oleic. Solid squares meant significant correlations (*p* < 0.05), blue squares were negative correlations, red squares were positive correlations.

Recently, the beneficial effect of IL-6 on liver metabolism has been demonstrated [31]. In our previous work, it was suggested that IL-6 has a positive effect on hepatocytes in the liver with NAFLD by blocking trans-signaling IL-6 and activating non-canonical signaling pathway NF-kB in patients with type 2 diabetes mellitus [22]. Blocking trans-signaling IL-6 by gp130 increased cells viability and increased *NF-kB1* gene expression in cell models steatohepatitis with tBHP/Oleic. NF-kB was a key activator of many proinflammatory genes and plays an important role in inflammation [32] and liver regeneration [33]. This fact is confirmed by the presence of correlation relationships in cell models with tBHP/Oleic: *NF-kB1* gene expression correlated positively with the percentage of live cells (r = 0.590) and negatively with the percentage of dead (r = −0.664) and apoptotic cells (r = 0.472) (*p* < 0.05) (Figure 7).

IL-6 has properties aimed at maintaining a normal mitochondrial population by increasing the activity of mitochondrial dynamics genes in hepatocytes under conditions of oxidative stress. Indeed, the addition of IL-6 enhanced the expression of *DNM1L, MFN2*, and *TFAM* genes in HepG2 cell cultures with tBHP/Oleic.

Disruption of mitochondrial dynamics is one of the most important links in the pathogenesis of NAFLD [34]. The predominance of mitochondrial division or fusion has pathological significance [35]. It is suggested that the predominance of mitochondrial division indicates cellular degradation [36]. IL-6 suppressed the expression of the *DNM1L* gene for mitochondrial division in tBHP/Oleic cell cultures.

Mitochondrial fusion is one of the most important processes by which the intracellular mitochondrial quality system can repair damaged mtDNA [37]. The increased *MFN2* gene expression we detected in the tBHP/Oleic model with the addition of IL-6 may indicate the compensatory nature of mitochondrial fusion as a protective mechanism against oxidative stress and lipid droplet accumulation in hepatocytes. In the tBHP/Oleic cell model, *MFN2* gene expression correlated positively with the percentage of live cells (r = 0.847) when IL-6 was added and negatively with the percentage of dead (r = −0.777) and apoptotic cells (r = −0.794) when IL-6 was added (*p* < 0.05) (Figure 7).

Thus, the addition of IL-6 contributed to the activation of mitochondrial dynamics, with mitochondrial fusion predominating to resist oxidative stress and fatty infiltration of hepatocytes.

We tested the effect of blocking the IL-6/IL-6Ra complex with soluble gp130 on cellular viability under oxidative stress [38]. Normally, the level of IL-6 in blood is extremely low (1–5 pg/mL), sIL-6R was detected in blood at concentrations of 40–70 ng/mL, and the soluble form of gp130 (sgp130) formed by alternative splicing is detected at concentrations of about 400 ng/mL [39]. The in vitro experiment used the concentrations of cytokines selected on the basis of their own studies in obese patients with type 2 diabetes in blood plasma, which were generally lower than those reported by other authors.

Gp130 increased cell viability twofold in HepG2 cultures with tBHP/Oleic. Addition of gp130 had a stimulatory effect on mitochondrial fission and fusion genes under conditions of oxidative stress. When oleic acid was added, mitochondrial division and replication genes were repressed and the mitochondrial fusion gene was activated to compensate for the toxic effects on cells. *MFN2* gene expression correlated with the percentage of live (r = 0.701), dead (r = −0.629), and apoptotic cells (r = −0.680) in HepG2 culture with oleic acid (*p* < 0.05) (Figure 7).

*DNM1L* gene expression in the tBHP/Oleic model correlated negatively with the percentage of live cells (r = −0.481) and positively with the percentage of dead cells (r = 0.209) (*p* < 0.05) (Figure 7). However, when gp130 was added to the tBHP/Oleic model, *DNM1L* gene expression decreased.

Under conditions of oxidative stress, mtDNA is damaged [40]. TFAM plays a central role in mtDNA metabolism [41]. Cells with a low amount of mtDNA have reduced *TFAM* expression compared to cells with a normal amount of mtDNA. This suggests a quantitative relationship between TFAM and mtDNA [42]. This is consistent with previously obtained data from obese patients: *TFAM* gene expression decreased in liver biopsies [22]; mtDNA copy number also decreased in patients with type 2 diabetes mellitus [36]. Addition of gp130 in tBHP/Oleic models increased *TFAM* gene expression, which is responsible for mitochondrial replication. It is possible that increased Tfam production, accompanied by increased mtDNA transcription/replication, mediates a reduction in oxidative damage to hepatocytes [43].

Sod is associated with mitochondrial replication activity [44]. Sod is an enzyme that catalyzes the decomposition of superoxide into molecular oxygen and hydrogen peroxide [45]. Sod-2 plays a critical role in the homeostasis of mitochondrial ROS [46]. ROS are suppressed by components of antioxidant defense systems. However, when the production of ROS exceeds the antioxidant capacity of cells, they cause further damage to mitochondria and other cellular components, setting the stage for oxidative stress and leading to damage to cells and tissues [47]. The expression of *SOD2* gene decreased in the cell model tBHP/Oleic compared to the control culture. Thus, the mechanisms of antioxidant protection and mitochondrial replication are linked and aim to reduce inflammation in the presence of oxidative damage.

**Figure 8:**
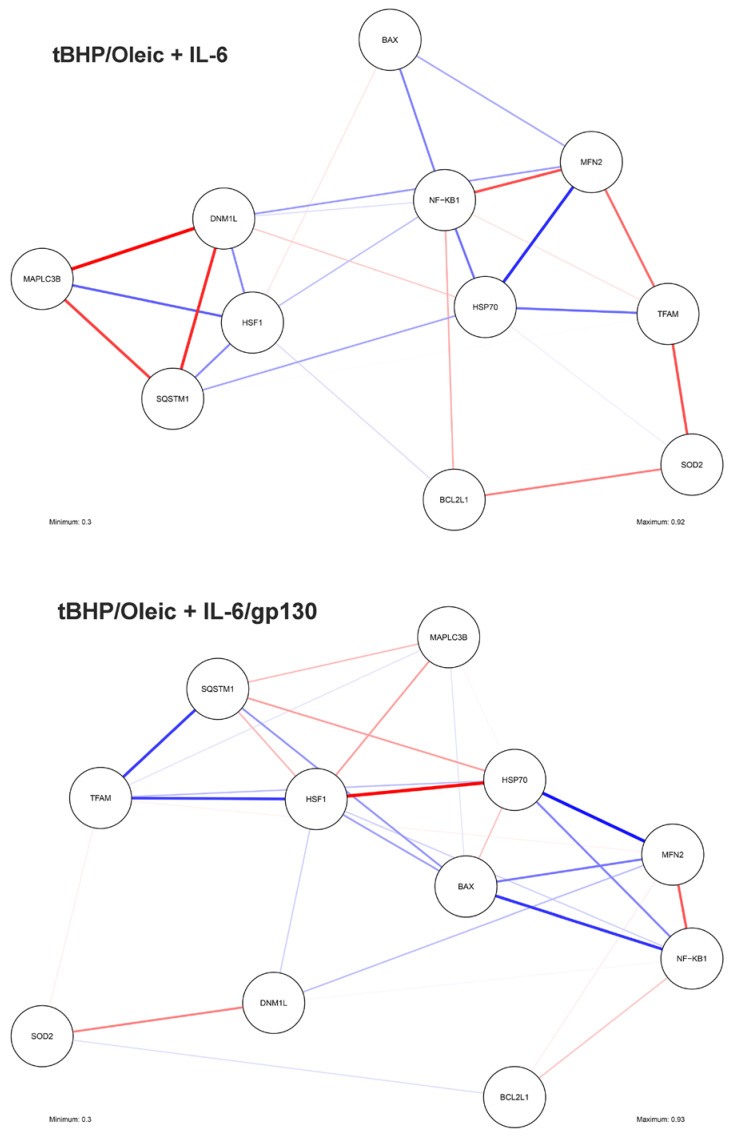
Correlations between gene expression in HepG2 with tBHP/Oleic. Blue lines are negative correlations, red lines are positive correlations. The line thickness indicates the strength of the correlation between the parameters.

The components of antioxidant defense systems and mitochondrial dynamics are interconnected. *TFAM* gene expression positively correlated with SOD2 gene expression (r = 0.496, *p* < 0.05) (Figure 8). *TFAM* may be involved in the reduction of ROS through increased antioxidant activity (including increased expression of *SOD2*), activation of 5′-adenosine monophosphate-activated protein kinase (AMPK) and mitochondrial dissociation, and regulation of membrane potential [48].

Heat shock proteins also protect cells from various stressors. Hsp70 is the most abundant and best conserved heat shock protein in the family. It functions as a chaperone that helps other proteins to adopt a native conformation or restore their function after misfolding, and is also involved in the selective degradation of oxidized proteins by the 20S proteasome [49]. When IL-6 and gp130 were added to the tBHP/Oleic cell model, *HSP70* gene expression and Hsp70 protein levels decreased compared to the control. In the tBHP/Oleic cell model, *HSP70* gene expression correlated with the percentage of live (r = −0.532), dead (r = 0.628), and apoptotic cells (r = 0.518) when IL-6 was added (*p* < 0.05). In the tBHP/Oleic cell model, *HSP70* gene expression correlated negatively with the percentage of live (r = − 0.547) and positively with the percentage of dead (r = 0.642) and apoptotic cells (r = 0.546) after addition of gp130. The data suggest that an increase in mitochondrial dynamics is sufficient to reduce the overproduction of ROS. This hypothesis is supported by the negative correlations we observed after addition of IL-6—*HSP70* and *MFN2* gene expression (r = −0.724), and *HSP70* and *DNM1L* gene expression (r = 0.425) (*p* < 0.05)—and the negative correlations we observed after addition of gp130—HSP70 and MFN2 gene expression (r = −0.879), and *HSP70* and *DNM1L* gene expression (r = 0.762) (*p* < 0.05) (Figure 8). However, the semiquantitative analysis of Hsp70 is not completely convincing. We hypothesize that most Hsp70 molecules can bind to damaged proteins to allow their refolding.

**Figure 9:**
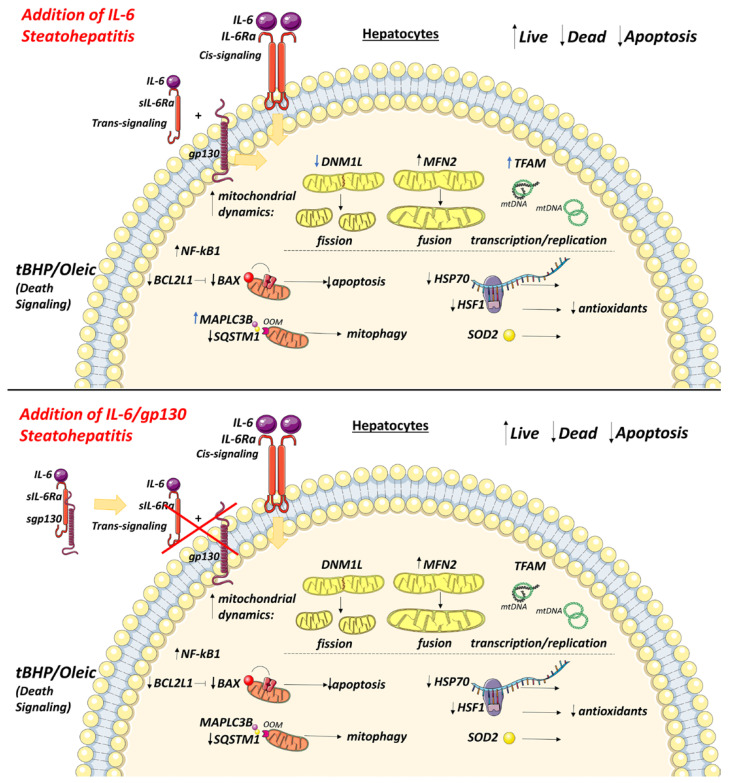
Visualization of the results of the study of cis- and trans-signaling of IL-6 in cellular models of steatohepatitis. The addition of IL-6 increased the expression of mitochondrial dynamics genes (*MFN2* and *TFAM*), and decreased the expression of the mitochondrial division gene (*DNM1L*) and apoptosis marker genes (*BCL2L1, BAX*), which led to an increase in the percentage of living cells in the steatohepatitis model. The addition of gp130 increased the expression of the mitochondrial fusion gene (*MFN2*), and decreased the expression of apoptosis (*BCL2L1, BAX*) and mitophagy (*SQSTM1*) marker genes, which led to an increase in the percentage of living cells in the steatohepatitis model. Addition of IL-6 or gp130 reduced the expression of cytoprotector genes (*HSF1* and *HSP70*) in HepG2 cell cultures with tBHP/Oleic.

Trans-signaling led to increased *TFAM* and *MAPLC3B*, and decreased *DNM1L* gene expression in HepG2 with tBHP/Oleic (Figure 9).

Management of IL-6 at the post-receptor level will allow a finer regulation of the processes of cell survival in steatohepatitis. Blocking IL-6 trans signaling promotes the activity of mitochondrial fusion genes and suppression of apoptosis and mitophagy genes in the steatohepatitis model.

It is known that NAFLD is a multicomponent disease, including disorders of lipid, carbohydrate, and energy metabolism. In this regard, further research should be directed to the study of trans-signaling in other circumstances of NAFLD.

## 5. Conclusions

The role of IL-6 in the pathogenesis of steatohepatitis depends on the type of signal transmission. Although activation of cis- and trans-IL-6 signaling led to an increase in cell viability in the HepG2 model of steatohepatitis, a number of differences were observed in the intracellular mechanism of action. Blocking trans-signaling of IL-6 led to the activation of the mitochondrial fusion gene and to a decrease in the activity of apoptosis and mitophagy marker genes. Numerous studies strongly suggest the potential for blocking IL-6 in the treatment of NAFLD. However, targeting blocking of IL-6 trans signaling could be an adjunctive therapy for NAFLD.

## Figures and Tables

**Table 1:** Sequences of primers and probes for PCR.

Gene	Sequence
*NF-kB1*	Forward: 5′-CAGGAAGATGTGGTGGACCA-3′ Reverse: 5′-AGGCCCGGCTCTGTCTAGTG-3′ Probe: 5′-FAM-GGCTGGAGGAGGCGGGCGTCTAAA-BHQ1- 3′
*HSF1*	Forward: 5′-AGAGAGAGACGACACGGAGTT-3′ Reverse: 5′-CTGTCCTGGCGGATCTTTATGT-3′ Probe: 5′-FAM-AGAGGAAAGTGACCAGTGTGTCCACCCT-BHQ1-3′
*SOD2*	Forward: 5′-CGTGGCTGTGGTGGCTTC-3′ Reverse: 5′-CGTGGTGCTTGCTGTGGT-3′ Probe: 5′-FAM-CCTCCCCGACCTGCCCTACGACTA-BHQ1-3′
*TFAM*	Forward: 5′-CGCTCCCCCTTCAGTTTTGT-3′ Reverse: 5′-TACCTGCCACTCCGCCCTAT-3′ Probe: 5′-FAM-CGAGGTGGTTTTCATCTGTCTTGGCA-BHQ1-3′
*HSP70*	Forward: 5′-CTTCGTGGAGGAGTTCAAGAGAAA-3′ Reverse: 5′-TAGAAGTCGATGCCCTCAAACAG-3′ Probe: 5′-FAM-AAGGACATCAGCCAGAACAAGCGAGCC-BHQ1-3′
*BAX*	Forward: 5′-AGTAACATGGAGCTGCAGAGGA-3′ Reverse: 5′-CCAGTTGAAGTTGCCGTCAGAA-3′ Probe: 5′-FAM-GATTGCCGCCGTGGACACAGACT-BHQ1-3′
*MFN2*	Forward: 5′-CCAGCGTCCCATCCCTCT -3′ Reverse: 5′-TCCACACCACTCCTCCAACA-3′ Probe: 5′ ACGGGCTCGCTCACCCAGGAG 3′
*DNM1L*	Forward: 5′-TCTGGAGGTGGTGGGGTTG-3′ Reverse: 5′-TGGGTTTTGATTTTTCTTCTGCTAAT-3′ Probe: 5′-FAM-ACCAACCACAGGCAACTGGAGAGGA-BHQ1-3′
*BCL2L1*	Forward: 5′-CCACCAGGAGAACCACTACA-3′ Reverse: 5′-CAGCCACAAACCCTTCCATA-3′ Probe: 5′-FAM-CACACCTCAGTTCCCTTGGCCTCA-BHQ1-3′
*MAP1LC3B*	Forward: 5′-TGCCTGTGTTGTTACGGAAAG-3′ Reverse: 5′-AGAAGGGAGTGTGTCTGAATGT-3′ Probe: 5′-FAM-ACTCTGGAGTACAGCGGGAGAAACACA-BHQ1-3′
*SQSTM1*	Forward: 5′-CCTGTCCCTGAAAGAGAAGATG-3′ Reverse: 5′-CAGAAAGTGTCAGAACCAGAGG-3′ Probe: 5′-FAM-CATGTGTAAGAACAATGCCAGGGCC-BHQ1-3′
*RPLPO*	Forward: 5′-GGCGACCTGGAAGTCCAACT-3′ Reverse: 5′-CCATCAGCACCACAGCCTTC-3′ Probe: 5′-Bgl635-ATCTGCTGCATCTGCTTGGAGCCCA-BHQ2-3′

## Data Availability

The data are available upon request from the corresponding authors.
